# The associations between self-reported depression, self-reported chronic inflammatory conditions and cognitive abilities in UK Biobank

**DOI:** 10.1016/j.eurpsy.2019.05.007

**Published:** 2019-08

**Authors:** Laura M. Lyall, Breda Cullen, Donald M. Lyall, Samuel P. Leighton, Stefan Siebert, Daniel J. Smith, Jonathan Cavanagh

**Affiliations:** a Institute of Health & Wellbeing, University of Glasgow, Glasgow, G12 8RZ, UK; b Institute of Infection, Immunity & Inflammation, University of Glasgow, Glasgow, G12 8TA, UK

**Keywords:** Depression, Inflammation, Inflammatory disease, Cognitive ability, Reaction time, Memory

## Abstract

**Background:**

Depression and chronic inflammatory medical conditions have been linked to impaired cognitive ability. However despite frequent comorbidity, their combined association with cognitive ability has rarely been examined.

**Methods:**

This study examined associations between self-reported depression and chronic inflammatory diseases and their interaction with cognitive performance in 456,748 participants of the UK Biobank, adjusting for sociodemographic and lifestyle factors. Numbers with available data ranged from 94,899 to 453,208 depending on the cognitive test.

**Results:**

Self-reported depression was associated with poorer performance compared to controls in several cognitive tests (fully adjusted models, reaction time: B = 6.08, 95% CI = 5.09, 7.07; pairs matching: incidence rate ratio = 1.02, 95% CI = 1.02, 1.03; Trail Making Test B: B = 1.37, 95% CI = 0.88, 1.87; Digit Symbol Substitution Test (DSST): B = −0.35, 95% CI = −0.44, −0.27). Self-reported chronic inflammatory conditions were associated with slower reaction time (B = 3.79, 95% CI = 2.81, 4.78) and lower DSST scores (B = −0.21, 95% CI = −0.30, −0.13). No interaction effects were observed.

**Discussion:**

In this large, population-based study we provide evidence of lower cognitive performance in both depression and a comprehensive category of chronic inflammatory conditions. Results are consistent with additive effects of both types of disorder on cognitive ability. Clinicians should be aware of such effects, particularly as cognitive impairment is linked to poorer disease outcomes and quality of life.

## 1 Introduction

Depression and certain medical conditions associated with chronic inflammation are associated with cognitive deficits [1,2], with inflammation proposed as a potential shared mechanism underlying these deficits [3]. In many chronic inflammatory conditions, however, potential cognitive consequences have not been studied, and despite frequent comorbidity of depression with inflammatory diseases, it is unclear whether such comorbidity is associated with worse cognitive outcomes relative to individuals with either depression or inflammatory disease alone.

Up to two thirds of patients suffering from depression show cognitive impairment in domains including executive function, episodic memory, attention and processing speed [1], with these deficits typically persisting in euthymia [4]. Cognitive impairment has also been observed in several chronic systemic or immune conditions, including rheumatoid arthritis (RA) and systemic lupus erythematosus (SLE) [2,5].

Chronic inflammation may partially explain the biology of major depression in certain groups of patients [3]. Increased peripheral inflammatory markers (e.g., Interleukin-6 (IL-6), C-reactive protein (CRP), tumour necrosis factor alpha (TNFα)) are associated with worse cognitive performance even in the absence of disease [6], consistent with a contribution of inflammation to the cognitive decline seen in depression [3]. Chronic inflammatory conditions such as RA and SLE are associated with higher prevalence of depression [7], and this comorbidity has been associated with worse disease outcomes, e.g., in remission rates, pain, and physical disability [8].

Studies of combined effects of depression and chronic inflammatory conditions on cognitive ability are however rare [9]. In the context of functioning and wellbeing, although effects have been small and inconsistent [10], some studies have reported interactions between depression and chronic disease status on various health-related outcomes. In line with the *‘amplification’* hypothesis of depression [11], larger negative effects of depression in the presence of chronic disease have been reported for quality of life [11] (arthritis, diabetes, hypertension), risk of falls [12] (cardiovascular disease, diabetes, arthritis), and activities of daily living [10] (diabetes).

Studies of combined effects of depression and chronic inflammatory conditions on cognitive ability are however rare. One study found that multiple sclerosis (MS) patients with and without depression did not differ on tests of verbal fluency, attention or verbal memory [13], while a recent systematic review found that depression in addition to diabetes was associated with reduced cognitive performance relative to diabetes without depression [9]. Aside from these studies, the potential for worse cognitive outcomes in comorbid depression and inflammatory disease, and the possibility that these types of disorders interact to have multiplicative effects on cognition has remained understudied, despite evidence of associations of cognitive decline in depression and inflammatory conditions with reduced quality of life and increased functional impairment [14]. We examined the association of self-reported depression and chronic inflammatory conditions, and their interaction, with cognitive ability in the UK Biobank cohort.

## 2 Methods

### 2.1 Participants

In 2006–2010, 502,543 adults aged 37–73 years were recruited to the UK Biobank and completed a baseline assessment at one of 22 UK assessment centres. Participants completed physical measures, demographic, lifestyle, health and mood questionnaires and several cognitive tests [15]. In 2015, participants were invited to complete further online cognitive tests. The 456,748 participants who completed at least one cognitive test at the baseline assessment and/or during the follow-up internet tests, and provided data on the sociodemographic characteristics included in fully and/or partially adjusted models were included in this study. Ethical approval for UK Biobank was obtained from the North West Multi-centre Research Ethics Committee (11/NW/03820).

### 2.2 Cognitive tests

Novel (non-standardised) tests of reasoning, pairs matching and reaction time were completed via a touchscreen at the baseline assessment, and online versions of standardised cognitive tests were completed an average of 5.82 years (SD = 0.86) later. At the baseline assessment, numerical memory and prospective memory were tested but are not examined here as only a subset of participants completed the numerical memory test, and the prospective memory test showed poor reliability [16].

#### 2.2.1 Baseline cognitive tests

##### 2.2.1.1 Reasoning

Participants answered up to 13 logical reasoning questions within a 2-minute time limit: scores range from 0 to 13. This test is referred to in UK Biobank documentation as ‘fluid intelligence’; however as the task is not standardised and also includes items testing crystallised intelligence, we refer to this as ‘reasoning’ [16].

##### 2.2.1.2 Pairs matching

Participants were presented with a grid of 12 cards and were asked to recall the positions of six matching pairs by selecting the overturned cards onscreen. Scores reflect visuospatial memory and consist of the number of errors before matching all six pairs (range: 0–146).

##### 2.2.1.3 Reaction time

Participants completed a computerised version of the card game ‘Snap’, where they were asked to press a button as quickly as possible when two cards presented onscreen matched. Scores consisted of the average time (milliseconds; ms) to give a correct response: incorrect responses were rejected, and reaction times of less than 100 ms and greater than 2000 ms were excluded. This task measures processing speed.

#### 2.2.2 Follow-up internet tests

##### 2.2.2.1 Trail making test (TMT)

Participants who accepted the invitation to the follow-up assessment completed an online, computerised version of the TMT [17]. Part A (TMT-A) involves clicking on 25 consecutively numbered circles in ascending order. In part B (TMT-B), participants again click on 25 circles in ascending order, this time alternating between numbers and letters. Scores for both parts consist of the time taken (seconds; s) to correctly click on all circles. The task involves processing speed and, in TMT-B, executive function [18]. For more information, see https://biobank.ctsu.ox.ac.uk/crystal/docs/trailmaking_doc.pdf.

##### 2.2.2.2 Digit symbol substitution test (DSST)

Participants were presented onscreen with a grid of eight symbols, each linked to a corresponding number. Using the number pad, they filled in the numbers corresponding to the symbols presented in boxes onscreen. Scores consisted of the total number of boxes correctly filled within a 2-minute time limit. This task is thought to involve processing speed, memory, attention, and executive function [[19], [20], [21]]. For more information, see https://biobank.ctsu.ox.ac.uk/crystal/docs/symdigsub_doc.pdf.

### 2.3 Health variables

In a nurse-led interview during the baseline assessment, participants were asked to report any medical conditions they had been diagnosed with by a doctor. Illnesses were recorded by the interviewer into a tree structure based on International Classification of Diseases 10^th^ Revision (ICD- 10) codes. In the current study, a list of conditions (excluding depression) that were reported by at least one participant and that are considered to be typically associated with chronic inflammation was derived and refined by three senior clinicians (JC, SS, SPL). Participants self-reporting a condition on this list (Table A1, Appendix A) were coded as having a ‘chronic inflammatory condition’. For secondary analyses, the list was subdivided into body systems based on ICD-10 chapters/sections: dermatological, digestive/abdominal, endocrine, infections, lymphoma/leukaemia, musculoskeletal/connective tissue, neurological, respiratory. See Table A1 (Appendix A) for numbers reporting each condition and category. Conditions that are typically chronic and/or recurrent were selected for the current study as the medical interview did not provide sufficient details on date/age of onset to establish if ‘acute’ conditions were present at the time of the assessment.

For primary analyses, classification of participants with a history of depression was based on self-report of a previous depression diagnosis during the nurse-led interview. In additional sensitivity analyses, we compared results when defining depression cases using criteria for probable recurrent moderate or severe major depressive disorder (MDD), based on responses to a brief mental health questionnaire (see [22] for details). Participants meeting criteria for bipolar disorder or single episode depression were excluded. In addition to the neuropsychiatric exclusions described in Table A2 (Appendix A), participants who self-reported depression during the medical interview were removed from the control group.

Participants who provided data on at least one cognitive test and on the sociodemographic covariates used in regression models were included and coded as self-reporting depression or not, and self-reporting one or more chronic inflammatory condition or not. This resulted in four groups: a) neither depression nor chronic inflammation (‘controls’); b) depression but no chronic inflammatory condition (‘depression only’); c) chronic inflammatory condition(s) but not depression (‘chronic inflammation only’); and d) both depression and chronic inflammatory condition(s) (‘depression plus inflammation’). Participants self-reporting certain other neuropsychiatric or neurological disorders or brain injury were excluded (Table A2, Appendix A). Numbers of participants in each group with available data on each cognitive test, as well as covariates included in partially and fully adjusted models, are summarised in Table A3 (Appendix A).

### 2.4 Sociodemographic and lifestyle variables

In a touchscreen questionnaire at baseline, participants were asked to report their age, sex, ethnicity, level of education, smoking status *(‘Never’, ‘Former’, ‘Current’*), and frequency of alcohol intake (Daily (*‘daily/almost daily’*), Regular (‘*1-2 or 3–4 times a week*’), Occasional (‘*special occasions only*’; ‘*1-3 times a month*’), or Never). For smoking and alcohol, ‘never’ was the reference category. Based on postcodes, Townsend deprivation scores were calculated: negative scores reflect greater affluence.

### 2.5 Analysis

A series of linear regression models were conducted. Negative binomial regression was employed for the pairs matching data, as scores consisted of skewed, overdispersed count data with many zero scores. In primary analyses, main effects of a) depression and b) chronic inflammatory conditions (across both levels of the second variable), and their interaction were examined. Main effects were calculated by effect coding binary depression and chronic inflammation variables as -1 (controls) and 1 (cases).

As we were interested in differences between those with comorbid depression and chronic inflammation vs. those with one type of condition only, simple effects were also tested in *a priori* contrasts. Each disease group (depression only, chronic inflammation only, and depression plus inflammation) was contrasted with the controls, and those with depression only and chronic inflammation only were compared with the group with both conditions.

For each model type, base models adjusted for age, sex, ethnicity (Caucasian vs. other) and deprivation (Townsend score). Fully adjusted models additionally adjusted for lifestyle variables: smoking status, frequency of alcohol intake and educational attainment (degree vs. no degree). For primary analyses (tests of main effects and interactions), a conservative significance threshold of *p* < 0.001 (FDR adjusted) [23] was used for all analyses to counter potential type-1 error associated with large numbers of contrasts: FDR adjustment was applied across all tests reported within each table. For descriptive statistics, a threshold of *p* <  0.001 (unadjusted) was applied. Robust standard errors were employed in all models.

## 3 Results

### 3.1 Descriptive characteristics

Sociodemographic characteristics and (unadjusted) cognitive test scores by group are displayed in Table 1, Table 2. The depression only group were younger, and the chronic inflammation only group were older, than the other groups. Most other characteristics followed a relatively linear worsening pattern from controls to depression plus inflammation: depression and/or chronic inflammatory conditions were associated with greater deprivation, reduced educational attainment, decreased proportion of never-smokers, and worse unadjusted cognitive performance.

**Table 1:** Descriptive statistics by self-reported depression and/or chronic inflammatory condition(s) groups.

	Controls (n = 361,240)	Depression Only (n = 19,133)	Chronic Inflammation Only (n = 70,715)	Depression plus Inflammation (n = 5,660)	Test statistic	p
Age, *M* (SD)	56.19 (8.13)	55.03 (7.86)*^†^	58.54 (7.68)*^†‡^	56.87 (7.54)*	1905.94	**<0.001**
Age at follow-up internet tests, *M* (SD)	61.75 (7.73)	60.22 (7.53)*^†^	63.51 (7.46)* ^†‡^	61.64 (7.46)	296.28	**<0.001**
Female, N (%)	198,507 (54.95)	12,991 (67.90)	33,089 (46.79)	3,522 (62.23)	3366.71	**<0.001**
White, N (%)	341,795 (94.62)	18,439 (96.37)	65,670 (92.87)	5,396 (95.34)	759.09	**<0.001**
Townsend score, *M* (SD)	−1.44 (3.01)	−0.91 (3.25)*^†^	−0.97 (3.23)*^†^	−0.16 (3.49)*	892.15	**<0.001**
Degree, N (%)						
Yes	120,259 (33.29)	6,015 (31.44)	19,503 (27.58)	1,429 (25.25)		
Missing data	6,834 (1.89)	169 (0.88)	1,271 (1.80)	73 (1.29)	1184.15	**<0.001**
Smoking status, N (%)						
Never	204,448 (56.60)	9,625 (50.31)	33,820 (47.84)	2,375 (41.96)		
Former	120,506 (33.36)	6,416 (33.53)	28,456 (40.24)	2,237 (39.52)		
Current	35,006 (9.69)	3,033 (15.85)	8,072 (11.41)	1,019 (18.00)		
Missing data	1,280 (0.35)	59 (0.31)	367 (0.52)	29 (0.51)	3138.17	**<0.001**
Alcohol intake frequency, N (%)						
Never	24,707 (6.84)	2,089 (10.92)	7,526 (10.64)	891 (15.74)		
Occasionally	78,000 (21.59)	5,152 (26.93)	17,788 (25.15)	1,779 (31.43)		
Regularly	183,452 (50.78)	8,102 (42.35)	31,580 (44.66)	2,058 (36.36)		
Daily/almost daily	74,808 (20.71)	3,759 (19.65)	13,753 (19.45)	921 (16.27)		
Missing data	273 (0.08)	31 (0.16)	68 (0.10)	11 (0.19)	3666.75	**<0.001**
Inflammatory conditions reported, *M* (SD)	--	--	1.12 (0.36)	1.18 (0.46)	149.77	**<0.001**

^*^Differs from controls. ^†^Differs from depression plus inflammation group. ^‡^Differs from depression only group. Test statistics are Analysis of Variance *F* values for continuous variables and χ^2^ for categorical variables. Pairwise contrasts are Tukey’s Honestly Significant Difference. BMI: body mass index; SD: standard deviation.

**Table 2:** Unadjusted cognitive test scores by group.

	Controls (n = 361,240)	Depression Only (n = 19,133)	Chronic Inflammation Only (n = 70,715)	Depression plus Inflammation (n = 5,660)	Test statistic	p
Reasoning, *M* (SD) (n = 154,402)	6.04 (2.15)	5.95 (2.20)^†^	5.79 (2.16)*^‡^	5.66 (2.18)*	107.44	**<0.001**
Missing	240,092	12,812	45,703	3,739		
Reaction time (ms), *M* (SD) (n = 452,513)	554.80 (114.16)	565.19 (124.21)*^†^	573.80 (124.58)*^†‡^	584.44 (134.66)*	634.42	**<0.001**
Missing	3,046	180	921	88		
Pairs matching, Median (Q1, Q3) (n = 453.208)	3 (2,6)	3 (2,6)	4 (2,6)	4 (2,6)	92.79	**<0.001**
Missing						
TMT-A (s), *M* (SD) (n = 95,822)	38.88 (14.87)	39.21 (15.08)	40.36 (15.31)*^‡^	41.19 (15.75)*	41.72	**<0.001**
Missing	282,639	15,506	57,985	4,796		
TMT-B (s), *M* (SD) (n = 95,820)	65.98 (25.20)	67.61 (26.46)^†^	69.99 (27.33)*^‡^	71.56 (28.09)*	102.89	**<0.001**
Missing	282,641	15,506	57,985	4,796		
DSST, *M* (SD) (n = 109,003	19.89 (5.19)	19.64 (5.35)^†^	18.86 (5.29)*^‡^	18.55 (5.07)*	181.86	**<0.001**
Missing	272,015	14,957	56,105	4,668		

^*^Differs from controls. ^†^Differs from depression plus inflammation group. ^‡^Differs from depression only group. Test statistics and pairwise comparisons are Kruskal-Wallis χ^2^ for pairs matching, and Analysis of Variance *F* values and Tukey’s Honestly Significant Difference for all others. DSST = Digit Symbol Substitution Test; ms = milliseconds; s = seconds. TMT = Trail Making Test. For N with available data for each cognitive test by group, see Table A3 (Appendix A).

### 3.2 Main effects and interactions

Main effects and interactions of depression and chronic inflammation are summarised in Table 3. A main effect of depression was present in partially adjusted models for all cognitive tests, with worse performance in the group reporting depression. In fully adjusted models, results were slightly attenuated in effect size, but all apart from reasoning and TMT-A remained significant at the FDR adjusted *p* < 0.001 threshold. In partially adjusted models, chronic inflammation was associated with worse performance on all tests apart from pairs matching and TMT-A. In fully adjusted models, main effects of chronic inflammation remained only for reaction time and DSST. Interactions between depression and chronic inflammation were not significant for any cognitive test in partially or fully adjusted models.

**Table 3:** Main effects and interactions for associations of depression and chronic inflammatory conditions with cognitive performance.

	Main effect of depression			Main effect of inflammation			Interaction		
	Beta (95% CI)	p	p FDR adj	Beta (95% CI)	p	p FDR adj	Beta (95% CI)	p	p FDR adj
Partially adjusted									
Reasoning (n = 154,402)	−0.06 (-0.09, -0.03)	**<0.001**	**<0.001**	−0.08 (-0.11, -0.05)	**<0.001**	**<0.001**	−0.01 (-0.04, 0.02)	0.470	0.584
Reaction time (n = 452,513)	6.61 (5.63, 7.59)	**<0.001**	**<0.001**	4.44 (3.45, 5.42)	**<0.001**	**<0.001**	0.38 (-0.60, 1.37)	0.443	0.584
Pairs matching* (n = 453,208)	1.02 (1.02, 1.03)	**<0.001**	**<0.001**	1.00 (0.99, 1.00)	0.291	0.436	1.00 (0.99, 1.01)	0.713	0.755
TMT-A (s) (n = 95,822)	0.56 (0.27, 0.85)	**<0.001**	**<0.001**	0.45 (0.16, 0.75)	0.002	0.004	0.11 (-0.18, 0.40)	0.456	0.584
TMT-B (s) (n = 95,820)	1.52 (1.02, 2.02)	**<0.001**	**<0.001**	0.98 (0.49, 1.48)	**<0.001**	**<0.001**	−0.03 (-0.53, 0.46)	0.891	0.891
DSST (n = 109,003)	−0.38 (-0.46, -0.30)	**<0.001**	**<0.001**	−0.26 (-0.34, -0.18)	**<0.001**	**<0.001**	−0.03 (-0.11, 0.05)	0.480	0.584
Fully adjusted									
Reasoning (n = 152,507)	−0.02 (-0.05, 0.002)	0.071	0.116	−0.03 (-0.06, -0.005)	0.021	0.036	−0.01 (-0.03, 0.02)	0.615	0.676
Reaction time (ms) (n = 442,670)	6.08 (5.09, 7.07)	**<0.001**	**<0.001**	3.79 (2.81, 4.78)	**<0.001**	**<0.001**	0.35 (-0.64, 1.34)	0.486	0.584
Pairs matching* (n = 446,541)	1.02 (1.02, 1.03)	**<0.001**	**<0.001**	1.00 (0.99, 1.00)	0.223	0.350	1.00 (1.00, 1.01)	0.620	0.676
TMT-A (s) (n = 94,901)	0.52 (0.22, 0.81)	**<0.001**	0.001	0.37 (0.08, 0.66)	0.014	0.024	0.11 (-0.18, 0.40)	0.472	0.584
TMT-B (s) (n = 94,899)	1.37 (0.88, 1.87)	**<0.001**	**<0.001**	0.71 (0.22, 1.21)	0.005	0.009	−0.06 (-0.55, 0.43)	0.812	0.835
DSST (n = 107,917)	−0.35 (-0.44, -0.27)	**<0.001**	**<0.001**	−0.21 (-0.30, -0.13)	**<0.001**	**<0.001**	−0.03 (-0.11, 0.05)	0.506	0.588

* Negative binomial regression for pairs matching models – reported coefficients are incident rate ratios. Effect coding was applied to both depression (-1 = no depression; 1 = depression) and inflammation (-1 = no chronic inflammatory conditions; 1 = chronic inflammation) groups. Partially adjusted model adjusted for age, sex, ethnicity, Townsend score. Fully adjusted model additionally adjusted for smoking status, frequency of alcohol intake and degree. CI = confidence interval; DSST = Digit Symbol Substitution Test; FDR = False Discovery Rate; ms = milliseconds; s = seconds; TMT: Trail Making Test.

### 3.3 A priori contrasts

*A priori* contrasts between each disease group and controls, and between those with depression plus inflammatory conditions vs. all other groups are reported in Table 4. Participants reporting depression only were slower on the reaction time and TMT-B tasks, made more pairs matching errors, and showed reduced DSST scores relative to controls in partially and fully adjusted models. Participants reporting only chronic inflammatory diseases showed deficits compared to controls in the reasoning, reaction time, TMT-A, TMT-B and DSST tasks in partially and fully adjusted models. Participants with both depression and inflammatory conditions were impaired relative to controls on all cognitive tests in partially adjusted models, and on all apart from reasoning and TMT-A in fully adjusted models. Participants with both depression and inflammatory conditions showed slower reaction times than those who reported depression only (partially and fully adjusted models). In partially and fully adjusted models, participants with both depression and inflammation showed poorer performance on the reaction time, pairs matching and DSST tasks compared to those with a chronic inflammatory condition(s) only.

**Table 4:** Associations between depression/inflammation status and cognitive test performance: *a priori* contrasts between groups.

	Depression Only vs. Control			Chronic Inflammation Only vs. Control			Depression plus Inflammation vs. Control			Depression plus Inflammation vs. Depression Only			Depression plus Inflammation vs. Inflammation Only		
	Beta (95% CI)	p	p FDR adj	Beta (95% CI)	p	p FDR adj	Beta (95% CI)	p	p FDR adj	Beta (95% CI)	p	p FDR adj	Beta (95% CI)	p	p FDR adj
Partially adjusted															
Reasoning (n = 154,402)	−0.10 (-0.16, -0.05)	**<0.001**	**<0.001**	−0.14 (-0.17, -0.11)	**<0.001**	**<0.001**	−0.29 (-0.38, -0.19)	**<0.001**	**<0.001**	−0.18 (-0.29, -0.07)	**<0.001**	0.001	−0.14 (-0.24, -0.05)	0.004	0.005
Reaction time (n = 452,513)	12.45 (10.72, 14.18)	**<0.001**	**<0.001**	8.11 (7.16, 9.07)	**<0.001**	**<0.001**	22.10 (18.66, 25.54)	**<0.001**	**<0.001**	9.65 (5.83, 13.47)	**<0.001**	**<0.001**	13.99 (10.45, 17.52)	**<0.001**	**<0.001**
Pairs matching* (n = 453,208)	1.04 (1.03, 1.06)	**<0.001**	**<0.001**	0.99 (0.98, 1.00)	0.007	0.009	1.04 (1.02, 1.06)	**<0.001**	**<0.001**	1.00 (0.97, 1.02)	0.720	0.720	1.05 (1.03, 1.07)	**<0.001**	**<0.001**
TMT-A (s) (n = 95,822)	0.90 (0.42, 1.38)	**<0.001**	**<0.001**	0.69 (0.41, 0.96)	**<0.001**	**<0.001**	2.03 (1.00, 3.06)	**<0.001**	**<0.001**	1.13 (0, 2.26)	0.050	0.058	1.34 (0.29, 2.40)	0.013	0.016
TMT-B (s) (n = 95,820)	3.11 (2.28, 3.93)	**<0.001**	**<0.001**	2.04 (1.56, 2.51)	**<0.001**	**<0.001**	5.01 (3.25, 6.76)	**<0.001**	**<0.001**	1.90 (-0.03, 3.82)	0.053	0.060	2.97 (1.16, 4.77)	0.001	0.002
DSST (n = 109,003)	−0.70 (-0.85, -0.55)	**<0.001**	**<0.001**	−0.46 (-0.54, -0.37)	**<0.001**	**<0.001**	−1.28 (-1.57, -0.99)	**<0.001**	**<0.001**	−0.58 (-0.9, -0.25)	**<0.001**	**<0.001**	−0.82 (-1.12, -0.52)	**<0.001**	**<0.001**
Fully adjusted															
Reasoning (n = 152,507)	−0.04 (-0.09, 0.02)	0.178	0.189	−0.05 (-0.08, -0.02)	**<0.001**	**<0.001**	−0.11 (-0.20, -0.02)	0.015	0.018	−0.08 (-0.18, 0.03)	0.145	0.158	−0.06 (-0.16, 0.03)	0.185	0.192
Reaction time (ms) (n = 442,670)	11.45 (9.73, 13.18)	**<0.001**	**<0.001**	6.89 (5.93, 7.84)	**<0.001**	**<0.001**	19.74 (16.28, 23.20)	**<0.001**	**<0.001**	8.29 (4.46, 12.12)	**<0.001**	**<0.001**	12.86 (9.31, 16.41)	**<0.001**	**<0.001**
Pairs matching* (n = 446,541)	1.04 (1.03, 1.06)	**<0.001**	**<0.001**	0.99 (0.98, 1.00)	0.001	0.002	1.04 (1.02, 1.06)	**<0.001**	**<0.001**	1.00 (0.97, 1.02)	0.707	0.719	1.05 (1.03, 1.07)	**<0.001**	**<0.001**
TMT-A (s) (n = 94,901)	0.82 (0.34, 1.30)	**<0.001**	0.001	0.52 (0.25, 0.80)	**<0.001**	**<0.001**	1.77 (0.73, 2.80)	**<0.001**	0.001	0.95 (-0.18, 2.08)	0.101	0.112	1.24 (0.18, 2.31)	0.021	0.025
TMT-B (s) (n = 94,899)	2.87 (2.05, 3.68)	**<0.001**	**<0.001**	1.55 (1.07, 2.02)	**<0.001**	**<0.001**	4.17 (2.43, 5.92)	**<0.001**	**<0.001**	1.31 (-0.60, 3.22)	0.180	0.189	2.63 (0.83, 4.42)	0.004	0.005
DSST (n = 107,917)	−0.65 (-0.80, -0.50)	**<0.001**	**<0.001**	−0.37 (-0.46, -0.29)	**<0.001**	**<0.001**	−1.13 (-1.42, -0.85)	**<0.001**	**<0.001**	−0.49 (-0.81, -0.16)	0.003	0.004	−0.76 (-1.06, -0.46)	**<0.001**	**<0.001**

* Negative binomial regression for pairs matching models – reported coefficients are incident rate ratios. Partially adjusted model adjusted for age, sex, ethnicity, Townsend score. Fully adjusted model additionally adjusted for smoking status, frequency of alcohol intake and degree. CI = confidence interval; DSST = Digit Symbol Substitution Test; FDR = False Discovery rate; ms = milliseconds; s = seconds; TMT = Trail Making Test.

### 3.4 Sensitivity analyses

To account for the possibility that the effects of chronic inflammation were driven by greater inflammatory disease load in the group with both depression and inflammation (Table 1), main effect and interaction analyses were repeated after exclusion of participants reporting more than one chronic inflammatory condition (Table A4, Appendix A). The significance and direction of main effects and interactions remained unchanged.

Analyses were also repeated after coding of depression using responses to baseline mental health questions instead of self-reported depression diagnoses (see [22]; Table A5, Appendix A). Main effects for depression and chronic inflammation were attenuated in terms of effect sizes and p values, likely due to reduced sample size. However, as in Section 3.2., depression was associated with more pairs matching errors and lower DSST scores in partially and fully adjusted models. Inflammation was associated with slower reaction time and lower DSST scores in partially and fully adjusted models. Chronic inflammation was also associated with *fewer* pairs matching errors relative to controls: this is likely linked to selection bias in the participants who reported inflammatory conditions and also completed the mental health questions and cognitive tests. As in primary analyses, no interactions attained significance.

### 3.5 Inflammatory disease category analyses

Main effects and interactions of depression and chronic inflammation were also examined separately for each category of inflammatory disease (see Table A6, Appendix A for N). Mutually exclusive categories were employed, with participants excluded from control, depression or inflammation groups if they reported inflammatory conditions in a second category. Fully adjusted main effect and interaction results are displayed for dermatological; digestive/abdominal; endocrine; infection; lymphoma/leukaemia; musculoskeletal/connective tissue; neurological; and respiratory conditions, respectively, in Tables A7-A14, Appendix A.

Main effects of depression were observed for musculoskeletal models for reaction time, pairs matching and DSST. Only a small number of main effects of depression were observed across the remaining models (reaction time for respiratory, endocrine and dermatological models and DSST for dermatological conditions). This is likely linked to reduced power, as participants with depression plus inflammatory conditions of another category were excluded from each sub-analysis. Due to similar loss of power, null results involving inflammation should also be interpreted with caution. Main effects of endocrine and neurological conditions were observed on reaction time, and main effects of neurological conditions on digit symbol scores were observed (Tables A9, A13, Appendix A). No interactions with depression were observed for any category.

## 4 Discussion

We report associations of both depression and chronic inflammatory medical conditions with cognitive performance in participants of the UK Biobank, independent of sociodemographic and lifestyle factors and education level. Depression was associated with impaired cognitive performance compared to controls in tasks involving processing speed (reaction time, TMT-B, DSST), visuospatial memory (pairs matching), and attentional control (TMT-B, DSST). Chronic inflammatory conditions were associated with slower reaction time and lower DSST scores. We did not find evidence of interactions between depression and chronic inflammation, either across all categories of inflammatory condition, or separately by disease category, suggesting associations between depression and cognitive abilities do not differ according to the presence of inflammatory conditions.

The cognitive impact of many of the included medical conditions, such as ulcerative colitis, eczema/dermatitis and gout, has rarely been investigated. This study therefore provides important evidence that cognitive impairments may be a feature of a wide range of conditions associated with heightened chronic inflammation. Associations with chronic inflammation were unchanged after excluding participants reporting more than one inflammatory condition, and so are unlikely to be driven by multimorbidity. Clinicians should be aware of associations of cognitive ability with chronic inflammation, particularly as impaired cognition is often associated with worse disease outcomes [2,24].

As heightened peripheral inflammation has been linked to depression and was a defining feature of the chronic conditions under study [25], findings are consistent with a role of inflammation in cognitive impairment. Several possible mechanisms may underlie this association. Increased pro-inflammatory proteins can cause changes in the status of neurotransmitters in the brain; expression of trophic growth factors and rate of neurogenesis; functioning of the hypothalamic-pituitary-adrenal (HPA) axis; and mechanisms of learning and memory. Together, these inflammatory-based mechanisms can result in a serotonin deficit and surplus of kynurenines within the brain, mechanistically important in both depression and cognitive impairment [3,26]. Also relevant for cognition, Cx3cr1+ monocytes and TNFα are important modulators of learning and memory via direct effects on neuronal dendritic spines [27,28].

Heightened peripheral inflammation, including in depressed patients, has been linked to reduced psychomotor speed [29], consistent with the associations of depression and inflammation with reaction time and DSST tasks here. Across all cognitive tests, reaction time most reliably demonstrated associations with depression and chronic inflammatory conditions. Power is a potential contributor here: the sample size for the reaction time task was higher than most others as this, along with pairs matching, was administered throughout the baseline period whereas only subsets of participants completed the other tests. Information processing speed is a potential endophenotypic marker for worse general cognitive abilities and structural brain ageing [30]. No associations with inflammation were however observed for pairs matching, which had similar sample size, despite previous reports of effects of peripheral inflammation or chronic inflammatory conditions on memory performance [2,31]. Lack of association of chronic inflammation with memory here could be linked to poor reliability and floor effects of the pairs matching task [16].

Overall, associations of depression with cognitive abilities were more likely to show significance, with larger effect sizes than for chronic inflammatory conditions. According to an inflammatory account of cognitive impairment, this stronger relationship with depression could reflect greater peripheral inflammation in depression. Alternatively, other factors, for example HPA axis dysregulation, circadian and/or sleep disturbances, physical inactivity or depressive symptoms, could have a greater influence on cognition in depression than in inflammatory conditions.

The lack of interaction effects implies additive effects of depression and chronic inflammatory conditions on cognitive performance. Consistent with this, participants with depression plus chronic inflammation showed worse cognitive performance in several domains compared to those with chronic inflammatory conditions alone, and showed slower reaction time compared to those with depression alone. These findings have important implications, as worse cognitive outcomes in those with both types of disorder may lead to poorer treatment adherence and therefore worse disease outcomes [32], as well as poorer functional independence and quality of life [14,24]. Clinicians should be aware that patients with chronic conditions who also suffer from depression may require additional support due to their potential for additional cognitive impairment.

For comparability of depression and chronic inflammation effects, in primary analyses, equivalent self-report measures were used for each. However, in sensitivity analyses, results were compared with those defining recurrent MDD using mental health questions asked at the baseline assessment (see [22]). Results were very similar, with associations of both depression and inflammation with impaired processing speed and association of depression with impaired memory.

A limitation of the current study is that it is not possible to distinguish between participants who reported no medical conditions because they do not recall any diagnoses, and those who declined to answer this question. However, as misclassification of individuals with existing conditions into the control group would be expected to bias results towards the null, particularly as individuals with cognitive impairment may be particularly likely to fail to recall/report a condition, spurious results are unlikely to arise from this. It is possible also that due to selection bias, patients reporting depression/inflammatory conditions who agreed to participate in the UK Biobank showed higher cognitive function than would be expected in the populations with these conditions – this could amount to a form of collider bias [33], but again, this would be expected to bias associations towards the null.

A further limitation is that we were not able to account for disease severity or for symptoms at the time of completing the cognitive tests, for depression or chronic inflammatory diseases. One possibility would have been to use participants’ self-reported medications to account for current disease status/severity, but as validity of self-reported medication differs widely by medication and is likely to differ between antidepressants and medications for chronic inflammatory conditions [34,35], this would have added additional confounds between the two types of condition. In addition, for many conditions there is no clear dominant form of medication. When primary care and prescription records become available through UK Biobank, it may be possible to more adequately control for medication and disease severity status.

Due to the cross-sectional nature of this study, it is not possible to infer the direction of causality. Baseline cognitive test data were collected at the same time as self-reported diagnosis data, with further online cognitive data collected several years later. It is therefore unknown whether cognitive decline preceded the onset of depressive or inflammatory disorders. Pre-existing cognitive dysfunction may be associated with higher risk of onset of depression or inflammatory conditions, e.g., via poorer coping strategies, lifestyle or environmental risk. Another possibility is that shared genetic risk factors are associated with both cognitive impairment and risk of physical and mental health problems. Longitudinal investigations will be of benefit in addressing the direction of causality.

Predictions that cognitive impairments in depression and chronic inflammatory conditions are partly driven by inflammatory mechanisms can be addressed more thoroughly in future studies which include data on inflammatory markers. For example in UK Biobank, associations between CRP and cognition, and any interactions with diagnoses (depression, inflammatory conditions) could be examined. Examination of associations of genetic risk for depression and chronic inflammatory disease (and their interaction) on cognitive outcomes will also be informative.

## 5 Conclusions

We provide evidence of inverse associations of self-reported depression and chronic inflammatory conditions with cognitive performance in a large population-based cohort. Self-reported depression was associated with slower processing speed and poorer visuospatial memory and attentional control. Participants reporting conditions typically associated with chronic inflammation had smaller deficits (compared to depression) in processing speed and attentional control. Interaction effects were not observed, suggesting the relationship of depression with cognitive function does not differ according to the presence/absence of chronic inflammatory conditions. The group with both depression and chronic inflammatory conditions showed the worst cognitive performance overall, including reduced processing speed and visuospatial memory compared to those with chronic inflammation only, and slower reaction time compared to those with depression only. Clinicians should be aware of the potential for cognitive deficits in those with depression and/or chronic inflammatory conditions, particularly as such deficits are likely to impact on medication adherence, functional independence and quality of life – individuals with depression in addition to chronic inflammatory diseases may be most at risk of such consequences.

## Funding

This research was conducted using the UK Biobank resource, under application 17689 (PI Lyall). UK Biobank was established by the Wellcome Trust, Medical Research Council, Department of Health, Scottish Government and Northwest Regional Development Agency. UK Biobank has also had funding from the Welsh Assembly Government and the British Heart Foundation. Data collection was funded by UK Biobank. JC acknowledges the support of The Sackler Trust and is part of the Wellcome Trust funded Neuroimmunology of Mood and Alzheimer’s consortium (104025/Z/14/Z) that includes collaboration with GSK, Lundbeck, Pfizer and Janssen & Janssen. DJS is supported by an Independent Investigator Award from the Brain and Behavior Research Foundation (21930) and a Lister Prize Fellowship (173096). DJS and LML are supported by an MRC Mental Health Data Pathfinder Award (reference MC_PC_17217).
